# Retraction: Can changes in implant macrogeometry accelerate the osseointegration process?: An *in vivo* experimental biomechanical and histological evaluations

**DOI:** 10.1371/journal.pone.0355242

**Published:** 2026-08-04

**Authors:** The PLOS One Editors

After this article [1] was published, concerns were raised regarding Figs 1-3, 5, 8, and 13-14; similarities between this article and other articles previously published by the same research group [2–3]; and the implant stability quotient (ISQ) values. Specifically:

- Articles [1,2] compare osseointegration outcomes for bone implants with the same macrogeometry surface feature (9 x 4 mm circular healing chambers) versus implants with a conventional surface and report similar:
  - Numbers of animals (20 in both [1] and [2]).
  - Numbers of implants (80, 2 per tibia, 4 per animal, with 6 control implants in both [1] and [2]).
  - Implant surface treatments (titanium oxide microparticle blasting plus conditioning with maleic acid).
  - Time points (21 and 28 days in [1], 15 and 30 days in [2]).
  - Measurements (implant stability quotient (ISQ), removal torque, histomorphological analysis).
  - Conclusions; specifically, that the macrogeometry surface feature improves early osseointegration.
- Neither [2] nor another article previously published by the same research group [3], reporting an *in vitro* investigation of an implant with the same macrogeometry surface feature as [1] and [2], were cited in [1].
- Despite both articles using the same type of implant and species of experimental animal, the ISQ values are lower in [2] than in [1].
- When flipped horizontally, Fig 13C of [1] appears similar to the upper half of Fig 11C of [2].
- Figs 5A and 14A of [1] appear similar to one another.
- The full implant schematics in Fig 1 of [1] appear similar to the implant schematics presented in Fig 3 of [3].
- The representative implant position image in Fig 2 of [1] appears similar to the upper tibia in Fig 5 of [2].
- The Materials and methods section states that animals were sacrificed at 3 or 4 weeks, however Fig 8 shows data for 3- and 6-week time points.

In response to the similarities between articles [1] and [2], the corresponding author stated that both [1] and [2] investigate the same implant macrogeometry versus a conventional design, and that they both share some experimental designs. The corresponding author further stated that both articles [1] and [2] were designed to investigate different questions, under different experimental parameters with different animals, and that they provide complementary data. They stated that [2] aims to evaluate whether implant macrogeometry or surface treatment plays a more decisive role in early osseointegration, whereas [1] focuses on the influence of surface macrogeometry in order to isolate the biological effects of the implant geometry and assess the performance of the implant at slightly later healing stages.

Regarding the lower ISQ values in [2] than in [1], the corresponding author stated these are due to the evaluation of different time points since implant stability undergoes dynamic changes throughout the osseointegration process, and are also due to different forms of data presentation as the values in [1] were aggregated per experimental unit (animal) whereas [2] presents individual implant values without aggregation.

In regard to the similarities between Fig 13C of [1] and Fig 11C of [2], the corresponding author stated that Fig 13C in [1] and Fig 11C in [2] are not the same, and that they are distinct histological sections obtained from different animal experiments and represent different implants and specimens.

The corresponding author stated that Figs 5A and 14A are the same image selected to represent the histomorphometric measurement area in Fig 5A, and to provide visual support for the quantitative data in the Results section of [1] in Fig 14A. The corresponding author also stated that Fig 1 of [1] and Fig 3 of [3] are the same implant design and macrogeometry created using the same software. They also stated that Fig 8 contains a typographic error: the bars labeled “6-weeks” should instead be labeled “4-weeks”. The *PLOS One* Editors consider these concerns resolved.

An independent member of the *PLOS One* Editorial Board reviewed articles [1] and [2], and the authors’ responses. They stated that the response that [1] assesses implant performance at a later stage is not clear, as the final time points in both articles are very similar, and are in fact slightly later for [2]. The *PLOS One* Editorial Board member also stated that the images presented in Fig 13C of [1] and Fig 11C of [2] appear to originate from the same histological section. Regarding the lower ISQ values in [2] than in [1], they stated that it is acceptable that ISQ values are different because of different evaluation time points, but that the explanation that the values were aggregated per experimental unit does not apply, and it is not clear how that would cause the measured, averaged values to be different.

In reviewing Table 1 and Fig 7, the P*LOS One* Editorial Board member noted that Time 1 should contain twice as many values as either Time 2 or Time 3 since all animals euthanized at T2 and T3 were also measured at the time of implant installation (T1). However, in the underlying data provided by the corresponding author, each time point contains the same number of values. The *PLOS One* Editorial Board member also raised concerns that the authors had not independently tested each time point against one another using appropriate non-parametric tests, and that tests for normality were not reported.

In response to the above concerns, the corresponding author stated that the ISQ measurements in Table 1 and Fig 7 are taken from two implants in a single animal that are not independent, and that they consider one animal to be one experimental unit. They also stated that, at T1, all 20 animals were evaluated, resulting in 40 raw ISQ measurements per group, however, the assessments at T2 and T3 were performed only after the animals were sacrificed at their respective time points (10 animals sacrificed at each of T2 and T3). The corresponding author stated that the Time 2 and Time 3 data consist of the mean values of two implants taken from one animal, whereas the Time 1 data consists of the mean values of four implants taken from two animals, hence the same number of values for all time points in the underlying data. They further stated the statistical analysis used in [1] was based on predefined comparisons according to the experimental design, taking into account the main effects and their interactions.

During post-publication discussions, the corresponding author provided the animal ethics approval issued by the University of Rio Verde for [1] and [2] where the named principal investigator is not listed as an author of [1]. The approved experimental protocols for [1] and [2] were not provided on editorial request and therefore PLOS have concerns that [1] and [2] may not be independent studies.

In light of the above concerns, the *PLOS One* Editors retract this article.

LPD agreed with the retraction. SAG, JAJ, TDDP, BAD, PM, and PNDA either did not respond directly or could not be reached.

Figs 1, 2, and 13C in [1] appear similar to content previously published in [3] (Fig 1) and [2] (Figs 2 and 13C) respectively under a CC BY license. No changes were made to the reused content. Figs 1, 2, and 13C are subject to the license that applies to the original articles; please provide due attribution to the original publications when referring to this content.

## References

[1] Gehrke SA, Júnior AJ, Pérez-Díaz L, do Prado TD, Dedavid BA, Mazon P, et al. RETRACTED: Can changes in implant macrogeometry accelerate the osseointegration process?: An *in vivo* experimental biomechanical and histological evaluations. PLoS One. 2020;15(5):e0233304. doi: 10.1371/journal.pone.0233304 32407416 PMC7224560

[2] Gehrke SA, Júnior AJ, Pérez-Díaz L, Treichel TLE, Dedavid BA, De Aza PN, et al. New implant macrogeometry to improve and accelerate the osseointegration: An in vivo experimental study. Applied Sciences. 2019;9(15):3181. doi: 10.3390/app9153181

[3] Gehrke SA, Pérez-Díaz L, Mazón P, De Aza PN. Biomechanical effects of a new macrogeometry design of dental implants: An in vitro experimental analysis. J Funct Biomater. 2019;10(4):47. doi: 10.3390/jfb10040047 31731451 PMC6963387

